# Risk stratification and diagnostic evaluation of patients found to have microscopic hematuria by their primary care providers

**DOI:** 10.1002/jgf2.740

**Published:** 2024-12-10

**Authors:** Clemens An, Jake Jeong, Cedrick Chiu, Evan Gaston, Amanda Kennedy, Kevan Sternberg

**Affiliations:** ^1^ The Robert Larner M.D. College of Medicine at the University of Vermont Burlington Vermont USA; ^2^ Washington University School of Medicine Saint Louis Missouri USA; ^3^ Department of Urology UMass Memorial Health Worcester Massachusetts USA; ^4^ Department of Medicine Quality Program The Robert Larner M.D. College of Medicine at the University of Vermont Burlington Vermont USA

**Keywords:** CT urogram, cystoscopy, guideline concordance, microscopic hematuria, patient outcomes, primary care providers, renal ultrasound

## Abstract

**Background:**

Our goal was to identify, and risk stratify primary care patients with microscopic hematuria (MH), describe the diagnostic evaluations they received, and determine whether the evaluations were consistent with the recommendations of the 2020 AUA/SUFU microscopic hematuria guidelines.

**Methods:**

A retrospective review of patients presenting to primary care clinics with a diagnosis of MH was performed. The patient risk category was determined based on the 2020 AUA/SUFU guidelines. Diagnostic strategies were recorded, and guideline concordance was determined. Descriptive statistics were generated to describe outcomes.

**Results:**

A total of 368 patients had a diagnosis of MH; 267/368 (72.6%) patients had all pertinent data available for risk stratification. One‐hundred and fifty‐six (58.4) patients were high‐risk and 55 (35.3%) had a urologic visit. Forty‐one of the 55 (75%) were diagnostically evaluated of which 13 (31.7%) were in‐line with guideline recommendations. Eighty‐two (30.7%) patients were at intermediate risk of which 33 (40.2%) had a urology visit. Of these 33 intermediate‐risk patients, 27 (81.8%) were diagnostically evaluated, five (18.5%) of which were in‐line with guideline recommendations. Twenty‐nine patients were low risk of which 4 (13.8%) had a urology visit. Three of the four patients seen by urology (75%) were evaluated with imaging studies and none received a cystoscopy.

**Conclusion:**

Almost 60% of the patients in our cohort were high‐risk according to the AUA/SUFU 2020 guidelines. Across all strata, the majority of patients lacked a urology visit and diagnostic evaluation consistent with guideline recommendations. Future efforts should ensure appropriate urologic referral and optimize initial diagnostic strategies for patients with MH.

## Graphical abstract

We conducted a retrospective review of patients who presented to their primary care outpatient clinics at a tertiary academic medical center and were diagnosed with microscopic hematuria. Patients were risk‐stratified based on the 2020 AUA/SUFU guidelines for microscopic hematuria. Urologic consultation and initial diagnostic strategies were recorded, and guideline concordance was determined. Descriptive statistics were generated to describe outcomes.

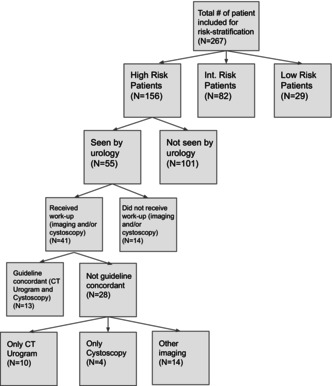

## 1 BACKGROUND

Microscopic hematuria (MH) is a common reason for primary care physicians (PCPs) to refer patients for urologic evaluation.^1^ Urinalysis (UA) is one of the most widely performed tests in the primary care setting and MH is frequently identified. In a 6‐year Kaiser Permanente database study, 3,742,348 UAs were conducted of which 552,119 (20%) were diagnostic for MH.^2^ In a systematic review by the American Urological Association of 80,000 patients, asymptomatic MH ranged from 2.4% to 31.1%.^3^ In another study, 1930 patients were evaluated for hematuria with a subgroup of 982 patients identified to have MH. From this subgroup, 92 (9.4%) of patients with MH were found to have cancer.^4^ The low prevalence of urologic malignancy identified because of MH makes it challenging to determine an optimal diagnostic approach.

While the evaluation of gross hematuria (GH) with cystoscopy, CT Urography (CTU), and urinary cytology is generally agreed upon, the diagnostic evaluation of MH is less well‐defined resulting in considerable practice variation. The previous version of the AUA MH guideline recommended the same workup for patients with MH and GH. However, because of the low risk of harboring malignancy in the setting of MH, the need for this evaluation strategy has been questioned.^3^ The imaging modality, specifically the need for CTU, presents the most controversy. Tan et al. performed a prospective observational study of 3556 patients with macroscopic or MH. The study demonstrated ultrasound's high sensitivity and negative predictive value of 85.7% and 99.9%, respectively for the detection of renal cancer. The authors also demonstrated that ultrasound (US) had limitations for the detection of upper tract urothelial cancer with a sensitivity of 14.3%. However, the extremely low incidence (0%) of upper tract urothelial cancer in those with MH, led to the conclusion that CTU could be safely replaced by US in these patients.^5^ In addition, Smith et al. identified 2138 patients with asymptomatic MH and found that US properly identified 3 of 3 patients with upper tract urothelial cancer demonstrating a sensitivity of 100%.^6^ These studies support that US can be considered as a reasonable diagnostic imaging modality for low‐risk patients who present with MH.

The goals of this study were to identify, and risk stratify a cohort of patients diagnosed with MH by their primary care provider. We then determined whether a urology visit occurred, the diagnostic strategies utilized, and whether these evaluations were concordant with the AUA/SUFU guidelines.

## 2 METHODS

**FIGURE 1:**
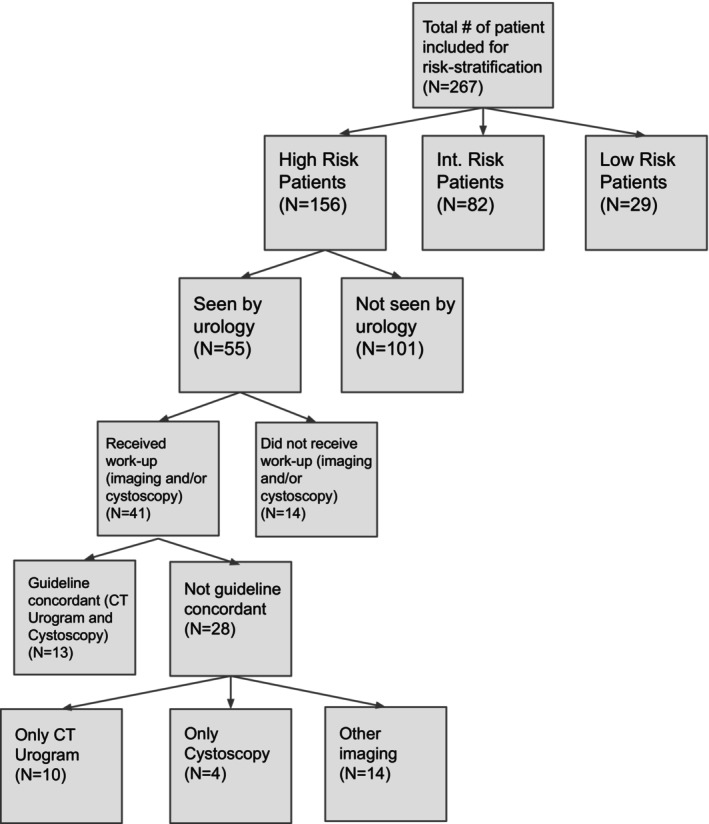
Study dataset flow chart.

A retrospective study was performed at The University of Vermont Medical Center; a 562‐bed academic medical center and affiliated primary care facilities. Eligible subjects included patients 18 years or older with a diagnosis of MH seen initially in the primary care setting between January 2020 and December 2021. Those with significant risk or strong clinical suspicion for malignancy were subsequently referred to urology at the main academic center hospital. UA was performed on the cohort for routine health monitoring at the discretion of the outpatient providers or to confirm the diagnosis of hematuria before initiating further diagnostic investigation. Patients without complete data to permit risk stratification were excluded (Figure 1).

Demographic information, insurance status, smoking pack years, urologic symptoms, family history, UA findings, referrals to urology, and diagnostic strategies were recorded from the electronic health record (Table 1). Data were collected and managed using REDCap electronic data capture tools hosted at The University of Vermont Referrals.^7^, ^8^ The patients within the dataset were initially assessed based on UA with microscopy results demonstrating the presence of MH (≥3 RBC/HPF).^9^ If a follow‐up UA was performed with negative results, this was noted as well. If a patient was referred to urology, the diagnostic tests obtained were collected. Evaluation modalities included were cystoscopies (cysto), US, CT urogram (CT uro), and any other type of CT scan (CT Other). Specific combinations of imaging were also considered (Table 2). Patients were stratified into risk groups according to the 2020 AUA/SUFU microscopic hematuria guidelines.^10^ If patients lacked the information regarding pack‐years smoked or UA with microscopy, they were not risk‐stratified and omitted from the analysis. Diagnosis for MH was recorded and stratified by group. This study was approved as exempt research by The University of Vermont Committee on Human Research (STUDY00001917).

**TABLE 1:** Study dataset characteristics.

Data element	Full data set (*n* = 368)		Risk‐calculated (*n* = 267)	
Age, median (IQR)	62.5	(50.7–70.9)	63.3	(50.7–70.9)
Gender identity: male, # (%)	126	34.2	82	30.7
Race: white, # (%)^a^	336	94.4	246	95.4
Ethnicity: Hispanic, # (%)^b^	8	2.3	6	2.3
Insurance, # (%)^c^				
Medicaid	29	7.9	19	7.1
Medicare	152	41.4	117	43.8
Commercial	184	50.1	131	49.1
None	2	0.5	0	0
Smoking: current, # (%)	25	6.8	16	6
Risk Factors: top 3, # (%)				
Kidney stones	49	13.3	37	13.9
BPH	15	4.1	11	4.1
Prostate cancer	10	2.7	5	1.9
Family history, # (%)				
Prostate cancer	48	13	35	13.1
Ureteral cancer	17	4.6	15	5.6
Bladder cancer	10	2.7	9	3.4
Renal cancer	5	1.4	0	0
Kidney cancer	1	0.3	1	0.4
Lynch syndrome	0	0	0	0
Kidney stones	1	0.3	0	0
BPH	0	0	0	0
Symptoms: top 3, # (%)				
Urinary frequency	82	22.3	57	21.4
Back pain	75	20.4	54	20.2
Dysuria	62	16.9	49	18.4
Referred to urology, # (%)	127	34.5	98	36.7

^a^ *n* = 356; ^b^ *n* = 356; ^c^ *n* = 367.

**TABLE 2:** Diagnostic tests performed based on risk‐stratified groups.

	Low risk (%)	Intermediate risk (%)	High risk (%)
Total	29 (10.9)	82 (30.7)	156 (58.4)
Cystoscopy (Cysto)	0 (0)	10 (12.2)	27 (17.3)
Ultrasound (US)	3 (10.3)	16 (19.5)	24 (15.4)
CT urogram (CT Uro)	2 (6.9)	15 (18.3)	36 (23.1)
CT of any other kind (CT Other)	9 (31.0)	27 (32.9)	60 (38.5)
Cysto and any imaging	0 (0)	10 (12.2)	25 (16)
Cysto and CT urogram	0 (0)	5 (6.1)	6 (3.8)
Cysto and CT Uro and CT Other	0 (0)	7 (8.5)	23 (14.7)
CT Any and US	2 (6.9)	5 (6.1)	10 (6.4)
No imaging	19 (65.5)	44 (53.7)	80 (51.3)
Guideline Concordance Rate	3 (10.3)	5 (6.1)	6 (3.8)

### 2.1 Statistical analysis

A descriptive analysis was performed. Eligible patients were divided into three separate groups: “Low Risk,” “Intermediate Risk,” and “High Risk” based on AUA/SUFU guidelines. The number and percentage of patients receiving urology consultation in each risk category were determined. The diagnostic evaluations received were then compared with the guideline recommendations to evaluate the number and percentage of concordant care for each risk category.

## 3 RESULTS

The initial cohort consisted of 368 patients with a diagnosis of MH. The average age of the cohort was 62.5 years and 243 (66.0%) were female. All eligible patients in the study are 18 years or older who received an initial diagnosis of MH in the outpatient setting. Additional study dataset characteristics are highlighted in Table 1. The 267/368 (72.6%) patients had all pertinent data available for risk stratification (Figure 1). Of these, 156 (58.4%) were considered high risk (HR), 82 (30.7%) were considered intermediate risk, and 29 (10.9%) were considered low risk.

Of the 267 patients who were included in the study were subsequently risk‐stratified, 185 were female and 82 were male. Of those who were referred to urology, 60 (32.4%) were female and 38 were (46.3%) male. Of those referred, only 92 (34.5%) were ultimately seen by urology. Of those seen by urology, 55 (59.8%) high‐risk, 33 intermediate‐risk (36.0%), and four low‐risk (4.3%), patients had a urologic visit following the MH diagnosis. The average age of the patients seen by urology was 62.1 years and that of those not seen was 62.4 years.

Of the 55 HR patients seen by urology, 41 (75%) were evaluated with imaging studies and/or cystoscopy. Of those, only 13 of the evaluations (31.7%) consisted of both CT urography and cystoscopy in‐line with guideline recommendations. Twenty‐eight (68.3%) of the diagnostic evaluations were guideline discordant: 10 (35.7%) with CT urogram alone, 4 (14.3%) cystoscopy alone, and 14 (50%) with other imaging studies (US and/or CT other). Of the 14 who received other imaging studies, 5 (35.7%) received only US, 4 (28.6%) received only CT other, and 5 (35.7%) received both US and CT other. Of these, 6 (3.8%) HR patient diagnoses for microhematuria were reported; 3 (50.0%) cases reported bladder cancer, 1 (16.7%) prostate cancer, 1 (16.7%) renal mass, and 1 (16.7%) nephrolithiasis.

Of the 33 intermediate‐risk patients seen by urology, 27 (81.8%) were evaluated with imaging studies and/or cystoscopy. Only 5 (18.5%) of the evaluations consisted of both renal US and cystoscopy in‐line with guideline recommendations. Twenty‐two (81.5%) of the diagnostic evaluations were guideline discordant: 5 (22.7%) received a cystoscopy with a CT imaging study, 2 (9.1%) received a renal US with a CT imaging study, 5 (22.7%) received only a renal US, and the remaining 10 (45.5%) patients received some combination of CT imaging and/or cystoscopy. Seven (8.5%) intermediate‐risk patients' diagnosis for microhematuria were reported. Three (42.9%) cases reported bladder cancer, 2 (28.6%) nephrolithiasis, 1 (14.3%) renal mass, and 1 (14.3%) renal cyst.

Of the 4 low‐risk patients seen by urology, three (75%) of them were evaluated with imaging studies. No cystoscopies were performed in this group of patients. One patient received a renal US in conjunction with a CT other. One patient received only CT other and one patient received only a CT urogram. Therefore, no low‐risk patients seen by urology received a diagnostic evaluation that was guideline‐concordant. 8 (27.6%) low‐risk patients' diagnosis for microhematuria were reported. Four patients were further evaluated by a urologist of which 1 (12.5%) bladder cancer, 1 (12.5%) renal cyst, and 2 (25%) nephrolithiasis cases were reported. Four patients were further evaluated by a primary care provider of which 3 (12.5%) nephrolithiasis and 1 (12.5%) musculoskeletal‐related hematuria cases were reported.

## 4 DISCUSSION

Most patients across all risk‐stratified groups diagnosed with MH by their PCP in our cohort did not have a urology visit or receive proper follow‐up screening. This is consistent with findings in the literature that demonstrate low urology referral rates for MH. Nieder et al. found that 36% of the patients presenting to their PCPs with MH were referred to urology.^10^ Another study by Yafi et al. found that only 48.6% of patients with two consecutive events of significant MH were recommended referral to urology.^11^ The findings of these studies highlight the lack of PCP guidance regarding referrals for this patient population.

The dilemma surrounding the diagnostic evaluation of MH involves the need to balance the potential negative sequela of CTU (radiation exposure, cost, and management of incidental findings) with the small but important concern of missing urologic malignancy.^12^ To address this concern, the updated AUA/SUFU guideline incorporates risk stratification for patients with MH. Specifically, patients with more risk factors for the presence of urologic malignancy based on age, RBC count on microscopy, smoking pack years, and history of GH warrant cystoscopy and CTU, while those at lower risk will be more appropriately evaluated with alternative imaging strategies such as renal ultrasound.

After risk stratification of our cohort, a large percentage of patients were classified as high‐risk and therefore warranted urologic consultation and workup with cystoscopy and CTU according to AUA guidelines. It is important to note that approximately 65% of the HR patients in our cohort did not see a urologist and even fewer had the recommended diagnostic evaluation. As the updated AUA guidelines have only been recently published, this is the first study to our knowledge that describes a population of patients with MH after risk stratification per guidelines. Across all strata, only 6.7% (18/267) of patients were given AUA guideline‐concordant care. Although the optimal treatment plans vary for each patient depending on unique independent factors, there is an alarming rate of incongruence from suggested AUA guidelines. One independent factor such as gender may have influenced the decision‐making process which may explain the difference in the referral rates by gender. Based on our cohort, the referral rate was 32.4% for women and 46.3% for men. One study demonstrated that women were referred to urologists less often and after a longer time since an initial presentation of hematuria when compared to men.^13^ It is certainly plausible that women are being erroneously treated for other diagnoses including urinary tract infections or postmenopausal bleeding, before being further evaluated by urology.^14^

Currently, there is ongoing debate regarding the ideal diagnostic evaluation for MH because of the very low rates of malignancy detection in this population of patients. In our cohort of intermediate and high‐risk patients, a 2.5% incidence of bladder cancer was found. Another study found a 2.7%, 0.4%, and 0% incidence of bladder, renal, and upper tract urothelial cancer, respectively.^5^ The previous AUA guidelines for the evaluation of MH recommended cystoscopy and CT urography for all patients with MH (3 or greater RBC/HPF).^3^ The goal of this approach was to minimize the chances of missing a urologic malignancy related to the MH diagnosis. The updated guidelines consider the overall low detection rate of malignancy and reserve CTU for those with the highest risk while low and intermediate‐risk patients would not receive CTU. The benefit of such an approach is to reduce costs, radiation exposure, and over‐identification of incidental findings that may require additional evaluation.^12^

Future quality initiatives in the form of educational sessions and risk stratification algorithms in electronic health records may be beneficial for PCPs to optimize the management of this patient population. Additionally, there is a need to reinforce guideline recommendations to current practicing urologists and PCPs to ensure consistency. With the increasingly aging general population and stagnation in the growth of urologists, PCPs, and urologists must be aware of MH guidelines to ensure that all patients with urologic concerns can be appropriately seen.

There are several limitations to our study. First is the retrospective design and its inherent drawbacks. Second, there were patient exclusions because of missing data preventing patient risk stratification. Third, many patients were missing a diagnostic cause of their microhematuria preventing a complete analysis of incidence. This can be attributed to numerous factors including poor follow‐up because of COVID protocols, lack of proper guideline adherence, and poor documentation. Patients who had hematuria and concurrent urinary tract infection symptoms were sometimes treated empirically without a formal visit which resulted in no formal documentation of a final diagnosis. Fourth, was the limited racial diversity in the population, primarily because of the institution's geographic location. Approximately 94.4% of participants identify as white, constraining our capacity to stratify the results by racial categories. Additionally, we were not able to identify the factors that led to a patient not receiving a urology visit. This could be because of numerous reasons in addition to the PCP not requesting a consult including the willingness of follow‐up care by patients, financial and other patient resource limitations, and strict COVID‐19 precautions. Sixth was the difficulty identifying which patients were recommended referral by PCPs versus those who were already being longitudinally followed by urology for another co‐morbidity. Therefore, a patient's imaging or cystoscopy may have been ordered as a result of another cause not related to MH. Additionally, guideline discordance in general likely resulted from various sources: incorrect imaging ordered from PCPs, varying practice preferences by urologists, and patient compliance. Therefore, causation cannot be determined by our study. Seventh, we do not have the clinical outcomes of the patients who were diagnostically evaluated or those who received follow‐up care at another health network system. However, this is not likely as our hospital is the only tertiary healthcare center for the entire state, and patients seeking care outside this health network would be very unlikely. Finally, because this was a study from one small population within the United States, it is not clear whether the breakdown of risk‐stratified patients mirrors the breakdown at the national level. Therefore, future work should look at risk‐stratification breakdown at a much larger level to assess this study's external validity.

## 5 CONCLUSION

A significant number of patients who present through the primary care setting with a diagnosis of MH are not receiving evaluations in accordance with the AUA/SUFU 2020 guidelines, especially a large number of high‐risk patients (156/267). Of the high‐risk patients who were diagnostically evaluated, about 1/3 ultimately received guideline‐concordant care. These current practice patterns risk missing the identification of urologic malignancy and can also lead to the inappropriate use of resources for those without a true diagnosis of MH. In summary, the results of this manuscript demonstrate an inherent discrepancy between guidelines and actual practice patterns for the workup and management of MH. A more coordinated care pathway between primary care services and urologists combined with education efforts is needed to raise guideline awareness and optimize the care of this patient population. We hope that these measures will ultimately help reduce the discrepancy between guidelines and actual practice partners.

## CONFLICT OF INTEREST STATEMENT

Authors declare no conflict of interests for this article.

## ETHICS STATEMENT

None.
